# Forecasting SPEI and SPI Drought Indices Using the Integrated Artificial Neural Networks

**DOI:** 10.1155/2016/3868519

**Published:** 2015-12-30

**Authors:** Petr Maca, Pavel Pech

**Affiliations:** Department of Water Resources and Environmental Modeling, Faculty of Environmental Sciences, Czech University of Life Sciences Prague, Kamycka 1176, Suchdol, 165 21 Prague 6, Czech Republic

## Abstract

The presented paper compares forecast of drought indices based on two different models of artificial neural networks. The first model is based on feedforward multilayer perceptron, sANN, and the second one is the integrated neural network model, hANN. The analyzed drought indices are the standardized precipitation index (SPI) and the standardized precipitation evaporation index (SPEI) and were derived for the period of 1948–2002 on two US catchments. The meteorological and hydrological data were obtained from MOPEX experiment. The training of both neural network models was made by the adaptive version of differential evolution, JADE. The comparison of models was based on six model performance measures. The results of drought indices forecast, explained by the values of four model performance indices, show that the integrated neural network model was superior to the feedforward multilayer perceptron with one hidden layer of neurons.

## 1. Introduction

Droughts are natural disasters and extreme climate events with large impact in different areas of economy, agriculture, water resources, tourism, and ecosystems. The reviews of significant drought events, their impacts, description, mitigation, and propagation in time are presented in detail in [1–3].

The drought indices are essential tools for explaining the severity of drought events. They are mainly represented in a form of time series and are used in drought modeling and forecasting [4]. The intercomparison of different drought indices connected with the development of forecasting tools was studied in large number of research studies [5–9].

The recent development of artificial neural networks (ANN) has a significant impact on the application of those techniques for the forecasting of drought indices. The ANN models are mostly represented by the nonlinear data driven black box modeling techniques. Empirical studies confirm that the multilayer perceptron (MLP) trained by the backpropagation algorithm is one of the most frequently studied ANN models, since it is a universal approximator [10–15].

The important direction in ANN research in water resources is the development and application of hybrid and integrated neural network models [16–19]. For example, Shamseldin and O'Connor [20] used the feedforward MLP for updating the outflow forecast. Huo et al. [16] developed the two versions of integrated ANN models and successfully applied them on monthly outflow forecast. The first version of integrated ANN models uses several outputs from several MLP models as inputs to final MLP; the second aggregates those outputs from several MLP models into one input to final MLP.

The main aims of the presented paper are to develop and apply several models of integrated neural networks for forecasting of drought indices and compare the integrated ANN models with the currently known models based on MLP. The rest of the paper is organized as follows.  Section 2 describes drought indices, architecture of tested neural network models, model performance measures, training method, and datasets.  Section 3 shows the results and discussion.  Section 4 concludes the paper.

## 2. Material and Methods

### 2.1. Drought Indices

The studied droughts were described using the two drought indices: the standardized precipitation index, SPI index [21–23], and the standardized precipitation evapotranspiration index, SPEI index [24, 25].

The SPI index is based on the evaluation of precipitation data. The precipitation data are linked to the selected probability distribution, which is further standardized using the normal distribution with zero mean and standard deviation of one. It is often expressed as a meteorological drought index [11], and it is used for the assessment of agricultural and hydrological droughts [21].

The estimation of SPI consists of the determination of probability distribution of analyzed precipitation data, the calculation of probabilities for measured precipitation data from cumulative distribution function of fitted probability distribution, and the application of the inverse of distribution function of normalized normal distribution on probabilities [21, 22].

The SPEI drought index is based on the precipitation and potential evapotranspiration data. The information about the potential evapotranspiration temperature is mostly derived using the temperature data. The SPEI index is expressed using the differences between precipitation and potential evapotranspiration. Its calculation technically follows the derivation of SPI index; the only difference is that instead of the precipitation time series the time series of the abovementioned differences are used [24, 25].

The estimation of SPI and SPEI drought indices was made using the R package [26]. The probability distribution of SPEI was expressed using the three-parameter log-logistic probability distribution; the SPI probability distribution was calculated using the Gamma distribution. The parameters were identified using the method of unbiased probability weighted moments [24, 25].

The SPI indicates the extremity of droughts. The SPI values split the range into extremely dry (SPI ≤ −2), severely dry (−2 < SPI ≤ −1.5), moderately dry (−1.5 < SPI ≤ −1), and near neutral conditions (−1.0 < SPI ≤ 1.0) [22, 23].

### 2.2. sANN Model

**Figure 1:**
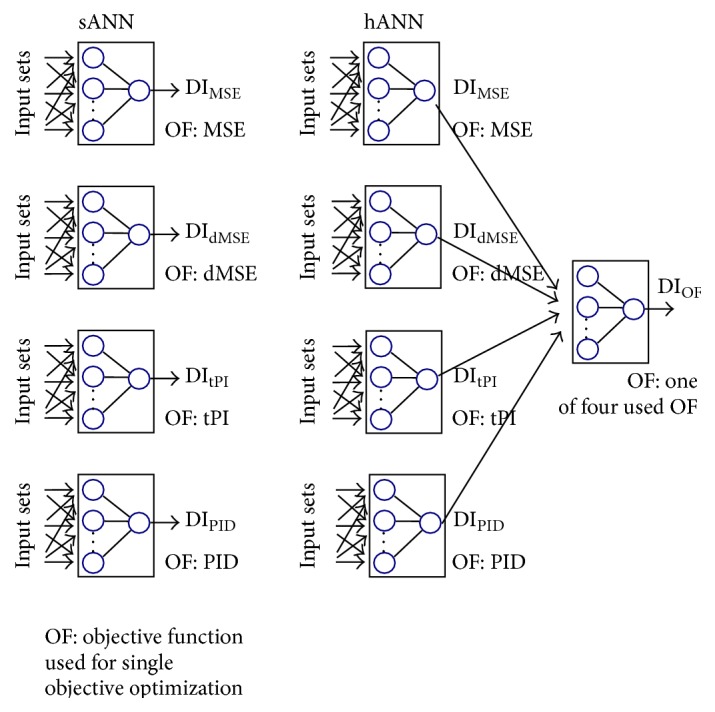
The scheme of tested ANN architectures, sANN, is based on single MLP; hANN is integrated ANN.

The architecture of the first analyzed neural network model was based on the feedforward multilayer perceptron with one hidden layer of neurons, sANN (the single ANN model; see Figure 1). This type of neural network architecture has been already applied on drought indices forecast [10, 11, 27].

The sANN model has the following mathematical formula:

(1) $$\begin{array}{c} {\text{DI}}_{f}=v_{0}^{\text{out}}+\overset{N_{\text{hd}}}{\underset{j=1}{\sum }}v_{j}^{\text{out}}f\left(\;v_{0j}^{\text{hd}}+\overset{N_{\text{in}}}{\underset{i=1}{\sum }}v_{ji}^{\text{hd}}x_{i}\;\right), \end{array}$$

where DI_*f*_ is a network output, that is, drought index forecast for a given time interval, *x*_*i*_ is network input for input layer neuron *i* normalized on the interval (0,1), *N*_in_ is the number of MLP inputs, *v*_*ji*_^hd^ is the weight of the connection between input *i* and hidden layer neuron *j*, *f*( ) is the activation function for all hidden layer neurons, *N*_hd_ is the number of hidden neurons, *v*_*j*_^out^ is the weight of the connection between the hidden neuron *j* and output neuron, and *v*_0*j*_^hd^, *v*_0_^out^ are biases of neurons [14, 28, 29].

The type of activation function of neurons in hidden layer was the RootSig [30, 31]. Its form is

(2) $$\begin{array}{c} y\left(\;a\;\right)=\frac{a}{1+\sqrt{1+a^{2}}}, \end{array}$$

with *a* = ∑_*i*=1_^*N*_in_^ *v*_*ji*_ *x*_*i*_ + *v*_0*j*_.

Since the neurons weights of sANN are unknown real parameters, their values were estimated using training algorithms and calibration and validation dataset of analyzed time series of drought indices. The number of hidden layer neurons was selected according to the current experience with drought indices forecast using the ANN models [10, 11, 27]. The presented analysis was focused on testing three sets of ANN models with different numbers of hidden layer neurons; *N*_hd_ = 4,6, 8.

### 2.3. hANN Model

The newly proposed hANN integrates five MLP (sANN) models. Figure 1 shows its scheme. The hANN is formed from two layers of sANN models. The first layer consists of four sANN. The second layer is formed from one sANN. Outputs of the first layer of sANN are inputs to the sANN in the second layer. The final forecast of the time series of selected drought index is obtained from the output from the last MLP. The tested architecture of hANN model was based on the integrated neural network model of Huo et al. [16].

The main enhancement lies in the inputs of the last MLP model. The inputs are obtained from four outputs from sANN models, which were trained according to the different neural network performance statistics: MSE, dMSE, tPI, and CI (see Section 2.4). Suggested approach combines the specific aspects of training sANN using different performance indices in one hybrid neural network. The last MLP is an error correction model or static updating model of drought index forecast [20, 32].

The unknown parameters of hANN are the real values of all sANN weights. Values of weights were estimated using the global optimization algorithm and calibration and validation datasets. We tested only those hANN models for which all five sANNs had the same number of neurons in the hidden layers. The training of hANN is explained in Section 2.5. The analyzed hANN models used the same inputs sets on four sANN in the first layer of hANN.

### 2.4. The Performance of ANN Models

The evaluations of ANN simulations of time series of drought indices for training and for validation datasets were based on the following statistics [33–35].

*Mean Absolute Error (MAE)*

(3) $$\begin{array}{c} \text{MAE}=\frac{1}{n}\overset{n}{\underset{t=1}{\sum }}\left|\;{\text{DI}}_{o}\left[\;t\;\right]-{\text{DI}}_{f}\left[\;t\;\right]\;\right|. \end{array}$$

*Mean Squared Error (MSE)*

(4) $$\begin{array}{c} \text{MSE}=\frac{1}{n}\overset{n}{\underset{t=1}{\sum }}{\left(\;{\text{DI}}_{o}\left[\;t\;\right]-{\text{DI}}_{f}\left[\;t\;\right]\;\right)}^{2}. \end{array}$$

*Means Squared Error in Derivatives (dMSE)*

(5) $$\begin{array}{c} \text{dMSE}=\frac{1}{n-1}\overset{n}{\underset{t=2}{\sum }}{\left(\;{\text{dDI}}_{o}\left[\;t\;\right]-{\text{dDI}}_{f}\left[\;t\;\right]\;\right)}^{2}. \end{array}$$

*Nash-Sutcliffe (NS) Efficiency*

(6) $$\begin{array}{c} \text{NS}=1-\frac{\sum_{i=t}^{n}{\left({\text{DI}}_{o}\left[t\right]-{\text{DI}}_{f}\left[t\right]\right)}^{2}}{\sum_{t=1}^{n}{\left({\text{DI}}_{o}\left[t\right]-\bar{{\text{DI}}_{o}}\right)}^{2}}. \end{array}$$

*Transformed Persistency Index (tPI)*

(7) $$\begin{array}{c} \text{tPI}=\frac{\sum_{t=1}^{n}{\left({\text{DI}}_{o}\left[t\right]-{\text{DI}}_{f}\left[t\right]\right)}^{2}}{\sum_{t=1}^{n}{\left({\text{DI}}_{o}\left[t\right]-{\text{DI}}_{o}\left[t-\text{LAG}\right]\right)}^{2}}. \end{array}$$

*Persistency Index (PI)*

(8) $$\begin{array}{c} \text{PI}=1-\text{tPI}. \end{array}$$

*Combined Index (CI) *

(9) $$\begin{array}{c} \text{CI}=0.85\text{tPI}+0.15\text{dMSE}. \end{array}$$

*Persistency Index 2 (PI2)*

(10) $$\begin{array}{c} \text{PI2}=1-\frac{\sum_{t=1}^{n}{\left({\text{DI}}_{o}\left[t\right]-{\text{DI}}_{{\text{ANN}}_{1}}\left[t\right]\right)}^{2}}{\sum_{t=1}^{n}{\left({\text{DI}}_{o}\left[t\right]-{\text{DI}}_{{\text{ANN}}_{2}}\left[t\right]\right)}^{2}}. \end{array}$$

*n* represents the total number of time intervals to be predicted, $\bar{{\text{DI}}_{o}}$ is the average of observed drought index DI_*o*_, dDI_*o*_[*t*] = DI_*o*_[*t*] − DI_*o*_[*t* − 1], and dDI_*f*_[*t*] = DI_*f*_[*t*] − DI_*f*_[*t* − 1], LAG is the time shift describing the last observed drought index DI_*o*_[*t* − LAG], and LAG is equal to two in the presented analysis. PI2 was applied on the comparison of forecast DI_ANN_1__ with DI_ANN_2__ made by two different neural network models.

### 2.5. The ANN Training Method

The training of tested sANN was based on solving inverse problems using the global optimization algorithm. The values of sANN parameters were found according to the minimization of performance indices MSE, dMSE, tPI, and CI. The performance indices were estimated on times series of analyzed drought indices. All sANNs were trained in batch mode. Only the single objective optimization methods were used [13, 36].

The training of hANN consisted of two steps. The first step was related to the training of four sANN models. Each sANN model had been trained using one of the four main objective functions: MSE, dMSE, tPI, and CI. The second step was based on the training of the last sANN. The fifth sANN was trained using one of the four objective functions and global optimization algorithm in batch mode. Training of one hANN was built on solving five single objective optimization problems [16].

The adaptive differential evolution, JADE, was applied as a main global optimization algorithm [37]. JADE is an adaptive version of differential evolution, which was developed by Storn and Price [38]. It is a nature inspired heuristics. The optimization process is based on the iterative work with population of models. Each population member is represented by the vector of its parameters. The differential evolution combines the mutation, crossover, and selection operators [39, 40].

The used adaptive mutation operator has the following formula:

(11) vkihd,out=vki−1hd,out+Fivp-besthd,out−vki−1hd,out+Fivr1i−1hd,out−vr2i−1hd,out.

The value of ANN weight *v*_*k*_^hd,out^ is changed during the *i*th iteration using *v*_*p*-best_^hd,out^ parameter, which is randomly selected from %*p* top models of population, *v*_*r*1_^hd,out^ and *v*_*r*2_^hd,out^ are weights of randomly selected models from population, and *F*_*i*_ is the mutation factor, which is adaptively adjusted using the Cauchy distribution. The top models in populations are those which have the best values of analyzed objective function in a given generation. The binomial crossover operator is controlled by the crossover probability CR_*i*_, which is automatically updated using the normal distribution. The detailed explanation of JADE parameter adaptation together with selection of *v*_*p*-best_^hd,out^ is presented in the work of Zhang and Sanderson [37].

### 2.6. The Dataset Description

We used for the drought indices neural network prediction the data obtained from two watersheds. The data were part of large dataset prepared within the MOPEX experiment framework [41, 42]. The MOPEX dataset provides the benchmark hydrological and meteorological data, which were explored in large number of environmentally oriented studies [43–47].

The first basin was Leaf River near Collins Mississippi with area 1924.36 km^2^ USGS ID-02472000 and the second was the Santa Ysabel Creek near Ramona California, 290.07 km^2^ USGS ID-11025500; both catchments are located in the USA. The original daily records were aggregated into the monthly time scale. We used the records from the period 1948–2002. The calibration period was formed from the period 1948–1975; the validation dataset consisted of the records from the period 1975–2002. The standard length of analyzed benchmark dataset was used in the presented study [42].

Table 1 shows the inputs for tested neural network models on both catchments. The forecasted output was DI[*t*] for both SPI and SPEI drought indices. *dT* was the monthly mean of averages of differences between daily maximum and minimum temperatures.

Although there were several derived automatic linear and nonlinear procedures of input selection for ANN models, the applied input selection procedure was iterative. Estimations of cross-correlation and autocorrelation of input time series were used for making the decision about the final tested input sets [48–50]. Since ANN models are capable of capturing the nonlinearities between the input and output data, we compared the ANN simulations, which were obtained using several combinations of different input variables with different memories. Table 1 presents the final list of the tested inputs.

The nonlinear transformation was applied on all ANN datasets. Its form was

(12) $$\begin{array}{c} D_{\mathrm{trans}}=1-\exp\left(\;-\gamma \left(\;D_{\text{orig}}+1.2\min\left(\;D_{\text{orig}}\;\right)\;\right)\;\right), \end{array}$$

with original data *D*_orig_, transformed *D*_trans⁡_, and minimum of untransformed data min⁡(*D*_orig_). This nonlinear transformation emphasises the low values, which are connected to severe drought events.

## 3. Results and Discussion

In our experiment, we analyzed for each inputs set 3 sANN architectures. They were formed by three different numbers of hidden neurons *N*_hd_ = 4,6, 8. All sANN and hANN models were calibrated 25 times using 4 objective functions, MSE, tPI, CI, and dMSE, and JADE optimizer. All five sANN models in one hANN had the same value of *N*_hd_. In total, we tested 1800 sANN models and 1800 hANN models.

The initial settings of JADE hyperparameters were similar for all ANN model runs. The population of models consisted of 20 × number of weights in sANN or hANN; the *v*_*p*-best_^hd,out^ was randomly selected from 45% percent of the best models in population. The best models in given generation were those which were sorted according to the values of objective function. The number of generations was 40. The selected values balanced the exploration and exploitation during the search process and helped to avoid the premature convergence of the population of the models. The hyperparameter *γ* of (12) was set to 0.15.

The results of ANN models trained using the dMSE were omitted in our presentation, since all models provided the worst results. However, outputs generated by sANN trained by the dMSE were inputs to the last sANN in all tested hANN models.

Since the Persistency Index (PI) is sensitive to timing error of forecast and enables the comparison of the simulation of drought indices with the naive model, formed by the last known information about drought index [33], we selected it as a main reference index.

### 3.1. The Forecast of SPI Index

The results of medians of model performance indices on SPI forecast are presented in Tables 2, 3, and 4. The medians were calculated for each set of 25 simulation runs. The SPI results are in several aspects similar to those of SPEI forecast.

When comparing the results of hANN with the results of sANN formed from single multilayer perceptron, the results of hANN were superior in terms of the medians of MAE, dMSE, MSE, and PI on calibration period for both catchments.

hANN models with nine inputs provided better SPI forecast according to the values of medians of PI index than models with six and three inputs. Similar recommendations on input datasets were confirmed in [10, 51].

The best models according to the medians of PI values were hANN models with 9-8-1 architecture with the last sANN trained by the MSE on Santa Ysabel Creek calibration dataset (PI = 0.80). The best values of median of Persistency Index (PI = 0.79) were obtained from hANN forecast on validation dataset using Leaf River dataset, 9-6-1 architecture, and tPI for optimization of last sANN (see Table 4).

**Figure 2:**
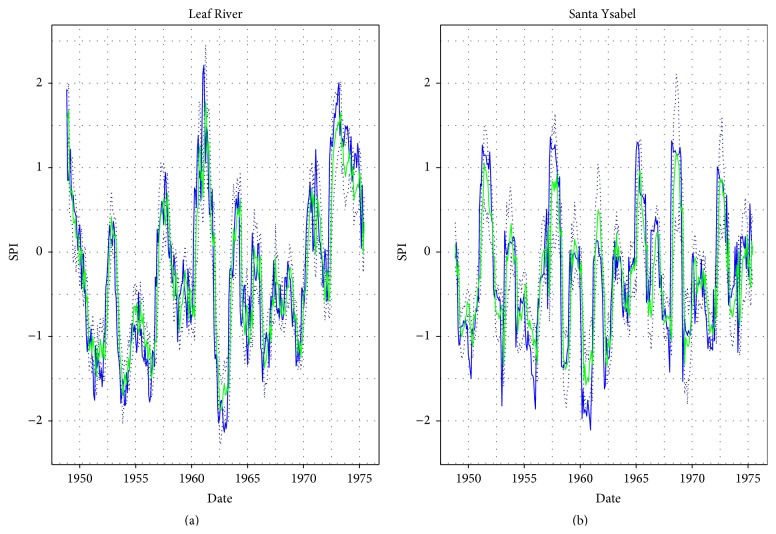
The calibration results of SPI forecast using hANN 9-8-1 trained on MSE: the blue line: observations, the green line: forecasted SPI medians, and the dotted lines: simulation error bounds.

**Figure 3:**
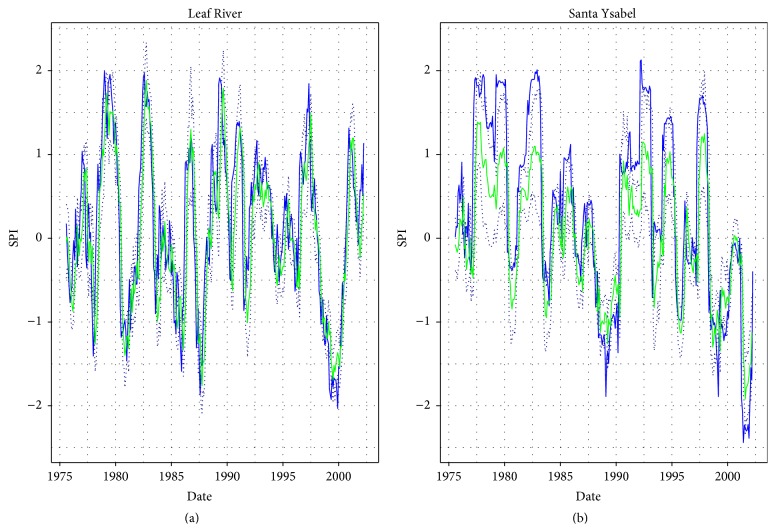
The validation results of SPI forecast using hANN, Leaf River 9-6-1 trained on PI, Santa Ysabel Creek 9-8-1 trained on PI. The blue line: observations, the green lines: forecasted SPI medians, and the dotted line: simulation error bounds.

Figures 2 and 3, respectively, show the forecast of SPI in calibration and validation, which were obtained using the hANN models with the highest values of medians of Persistency Index.

Results of PI2 index for the models with 9-*N*_hd_-1 architecture show that 9-8-1 architecture was superior for SPI forecast on validation datasets for both analyzed catchments (see Table 5). The comparison of calibration results shows that the 9-6-1 architecture has the highest values of PI2 on Santa Ysabel Creek dataset, and 9-8-1 has highest values of PI2 on Leaf River calibration dataset.

Table 6 shows the results of PI2 index, which were calculated using the results of hANN with 9-*N*_hd_-1 architecture. Values of PI2 enable us to compare the tested ANN models according to the performance of the optimization function, which were applied on training of the last MLP. The hANN models with the last sANN trained by the tPI were superior to hANN with last sANN trained using MSE or CI on calibration and validation Leaf River datasets.

The Santa Ysabel Creek datasets show that the best results were obtained by the tPI optimization for calibration of the last sANN of hANN models according to PI2. The values of PI2 for validation dataset show that hANN with the last sANN trained using the MSE provided better simulation results than the remaining hANN models (see Table 6).

### 3.2. The Forecast of SPEI Index

The mean performance of neural network models was explained using the medians of model evaluation metrics. The medians were obtained from the results of 25 runs on each basin for each ANN model architecture. Tables 7, 8, and 9 show the results of the evaluations of SPEI forecasts using the medians of MAE, MSE, dMSE, NS, and PI.

The integrated hANN models were superior to single multilayer perceptron models sANN in terms of the best values of medians of performance indices MAE, MSE, dMSE, and PI for both catchments. One of the exceptions can be found in Leaf River dataset: the NS of the single MLP for 3-8-1 trained using MSE on calibration and validation periods. However, this sANN model with the highest values of NS did not produce the highest values of PI (see the calibration period for 3-8-1 hANN and 3-8-1 sANN for Leaf River in Table 7).

hANN model results obtained from nine SPEI inputs were superior to the results obtained from ANN models with three and six inputs. Simulation results from hANN models with three inputs were superior in terms of PI to hANN models with six inputs in the first layer of sANN. Incorporation of other information into the ANN inputs did not improve the SPEI forecast.

**Figure 4:**
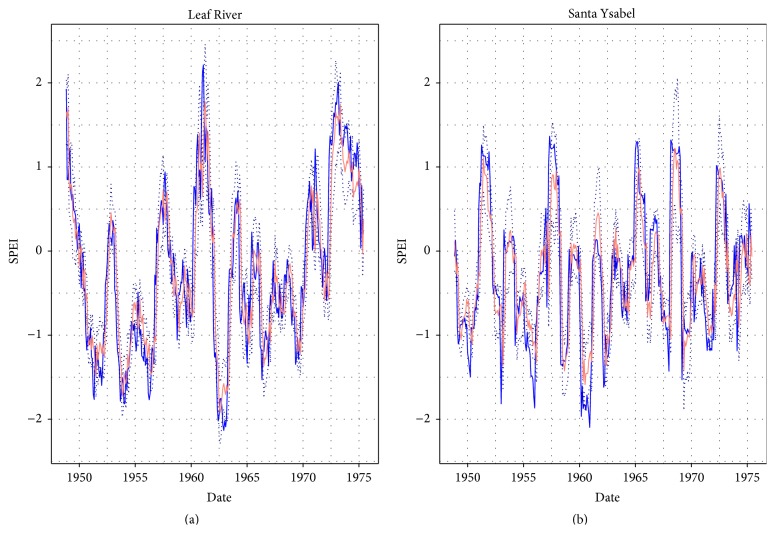
The calibration results of SPEI forecast using hANN: the blue line: observations, the salmon line: forecasted SPEI medians, and the dotted lines: simulation error bounds.

**Figure 5:**
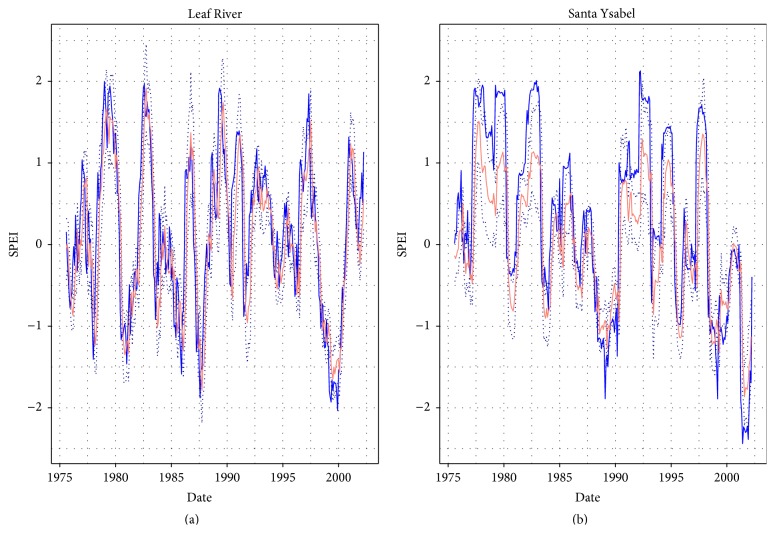
The validation results of SPEI forecast using hANN: the blue line: observations, the salmon line: forecasted SPEI medians, and the dotted lines: simulation error bounds.

The hANN models with 9-8-1 and 9-6-1 sANN architectures trained on tPI index were superior in terms of the values of PI for both catchments for calibration results. The calibration results of the best hANN models trained on tPI for both catchments are shown in Figure 4. The best simulation results according to the medians of PI were obtained for hANN on sANN architectures 9-8-1, 9-6-1 trained on tPI and MSE for both basins. The time series are shown in Figure 5.

When comparing hANN architectures with 9 inputs, the best models according to the PI2 were those with 6 hidden layer neurons in calibration of Leaf River dataset, while on validation data the best PI2 values were obtained from hANN modes with eight hidden layer neurons. On Santa Ysabel datasets, the models with best PI2 indices were hANN with 8 hidden layer neurons for calibration, while hANN models with 6 hidden neurons were superior to the validation dataset (see Table 10).

Table 11 shows the comparison of the influence of different optimization functions on the calibration and validation of hANN models with nine inputs. The optimization based on MSE was capable of providing better hANN models than the optimizations which used tPI and CI, on Leaf River dataset. The tPI optimization function enabled us to find hANN models, which had had better PI2 values in Santa Ysabel datasets. Note that the differences between PI2 for tPI and MSE are very small.

### 3.3. Discussion

The results of our computational experiment show the high similarities of values of SPI and SPEI drought indices. The values of correlation coefficients between the SPEI and SPI values were 0.98 for Leaf River dataset and 0.99 for Santa Ysabel dataset. Small differences between both drought indices reflect the fact that the temperatures trends were not apparent in both analyzed datasets [24, 25].

We used the single multilayer perceptron model as a main benchmark model for hANN. This model showed its simulation abilities and was compared with other forecasting techniques, for example, ARIMA models [11, 13, 27].

The comparison of ANN and hANN clearly confirms the finding which was made by Shamseldin and O'Connor [20] and Goswami et al. [32]. It shows the benefits of newly tested neural network model. The updating of simulated values of drought indices using the additional MLP was in all cases successful in terms of the improvement of SPEI and SPI drought forecast according to PI values. Similar overall benefits of the integrated neural network models were confirmed by [16] on simulations of monthly flows.

Our computational exercise also confirms the improvements of hANN drought forecast in terms of correcting the time shift error [11, 34, 35]. The tested hybrid neural network models decreased the overall time shift error in terms of dMSE values. However, the differences between hANN models trained on CI, designed to correct the time shift error, did not show significant improvements over the hANN trained on MSE or tPI.

The increased accuracy of drought index forecast was also influenced by using a model with the higher number of parameters. The simplest sANN with 3 inputs and 4 hidden layer neurons had 21 parameters, while the hANN with 9 inputs and 8 hidden layer neurons had 405 parameters. The high number of parameters may limit the application of hANN model in the case where other parsimonious models with similar simulation performances are available.

## 4. Conclusions

We analyzed the forecast of two drought indices, SPEI and SPI, using two types of neural network models. The first model was based on the feedforward neural network with three layers of neurons. The second one integrates the drought forecasts from five single multilayer perceptrons trained by the four different performance measures into the hybrid integrated neural network.

The SPEI and SPI neural network forecast was based on the data obtained from the period 1948–2002 from two US watersheds. The analyzed data were collected under MOPEX framework.

When evaluating the ANN models performance, the results of four from five model performance indices show that hybrid ANN models were superior to the single MLP models.

When comparing three different input sets on the SPEI and SPI forecast, the input sets with nine lagged monthly values of SPEI and SPI indices were superior. Adding the other types of inputs did not improve the results of neural network forecast.

The tested hANN and sANN models were trained using adaptive differential evolution. The nature inspired global optimization algorithm was capable of successfully training neural networks models. The optimization was based on four functional relationships describing model performance: MSE, dMSE, tPI, and CI indices. The worst training results were obtained with ANN models based on dMSE.

When comparing hANN models according to the number of neurons in the hidden layer, two neural network architectures, 9-6-1 and 9-8-1, generated the highest values of PI2 on SPEI and SPI forecast. Also when evaluating the influence of different optimization functions on hANN performance using PI2, the tPI and MSE neural network performance functions were superior to dMSE and CI.

Although SPEI and SPI indices are using the precipitation data and have some degree of similarity, the best predictions were obtained using the different combination of neural network model and training and training criteria.

## Figures and Tables

**Table 1:** The inputs for all tested neural network models.

Input set	Number of inputs	Input variables
3-*N*_hd_-1	3	DI[*t* − *i*] for *i* = 2,3, 4
6-*N*_hd_-1	6	DI[*t* − *i*] and *dT*[*t* − *i*] for *i* = 2,3, 4
9-*N*_hd_-1	9	DI[*t* − *i*] for *i* = 2,3,…, 10

**Table 2:** The medians of performance metrics for MLP architecture 3-*N*_hd_-1 on SPI forecast for sANN_TA and hANN_TA; TA shows the training error function, and the best models are marked with bold fonts.

	Calibration period					Validation period				
	MAE	MSE	dMSE	NS	PI	MAE	MSE	dMSE	NS	PI
*Leaf River*										
3-4-1										
sANN_MSE	3.88*E* − 01	2.56*E* − 01	1.85*E* − 01	7.45*E* − 01	−7.57*E* − 02	4.40*E* − 01	3.05*E* − 01	1.68*E* − 01	6.36*E* − 01	−1.55*E* − 01
sANN_tPI	3.90*E* − 01	2.57*E* − 01	1.90*E* − 01	7.45*E* − 01	−7.72*E* − 02	4.32*E* − 01	2.99*E* − 01	1.72*E* − 01	6.44*E* − 01	−1.32*E* − 01
sANN_PID	3.86*E* − 01	2.50*E* − 01	1.77*E* − 01	7.51*E* − 01	−4.95*E* − 02	4.31*E* − 01	2.93*E* − 01	1.67*E* − 01	6.50*E* − 01	−1.12*E* − 01
hANN_MSE	3.66*E* − 01	2.27*E* − 01	1.55*E* − 01	7.38*E* − 01	4.80*E* − 02	4.00*E* − 01	2.55*E* − 01	1.47*E* − 01	6.40*E* − 01	3.35*E* − 02
hANN_tPI	3.67*E* − 01	2.26*E* − 01	1.50*E* − 01	7.44*E* − 01	5.15*E* − 02	3.96*E* − 01	2.49*E* − 01	1.44**E** − 01	6.27*E* − 01	5.49*E* − 02
hANN_PID	3.64*E* − 01	2.25*E* − 01	1.69*E* − 01	7.39*E* − 01	5.55*E* − 02	3.94*E* − 01	2.48*E* − 01	1.52*E* − 01	6.20*E* − 01	6.15*E* − 02
3-6-1										
sANN_MSE	4.08*E* − 01	2.71*E* − 01	2.02*E* − 01	7.31*E* − 01	−1.36*E* − 01	4.24*E* − 01	2.83*E* − 01	1.69*E* − 01	6.62*E* − 01	−7.47*E* − 02
sANN_tPI	3.81*E* − 01	2.49*E* − 01	1.90*E* − 01	7.53*E* − 01	−4.34*E* − 02	4.21*E* − 01	2.81*E* − 01	1.74*E* − 01	6.65*E* − 01	−6.39*E* − 02
sANN_PID	3.84*E* − 01	2.47*E* − 01	1.85*E* − 01	7.55*E* − 01	−3.59*E* − 02	4.24*E* − 01	2.85*E* − 01	1.69*E* − 01	6.60*E* − 01	−7.91*E* − 02
hANN_MSE	3.64*E* − 01	2.23**E** − 01	1.62*E* − 01	7.51*E* − 01	6.39*E* − 02	3.88*E* − 01	2.42*E* − 01	1.54*E* − 01	6.30*E* − 01	8.24*E* − 02
hANN_tPI	3.63**E** − 01	2.23**E** − 01	1.48**E** − 01	7.46*E* − 01	6.32*E* − 02	3.86*E* − 01	2.34**E** − 01	1.45*E* − 01	6.25*E* − 01	1.14**E** − 01
hANN_PID	3.64*E* − 01	2.24*E* − 01	1.59*E* − 01	7.51*E* − 01	5.94*E* − 02	3.89*E* − 01	2.39*E* − 01	1.54*E* − 01	6.33*E* − 01	9.33*E* − 02
3-8-1										
sANN_MSE	3.74*E* − 01	2.32*E* − 01	2.07*E* − 01	7.60*E* − 01	2.71*e* − 02	4.10*E* − 01	2.66*E* − 01	1.84*E* − 01	6.83**E** − 01	−6.75*E* − 03
sANN_tPI	3.76*E* − 01	2.40*E* − 01	2.08*E* − 01	7.61*E* − 01	−7.60*E* − 03	4.14*E* − 01	2.73*E* − 01	1.85*E* − 01	6.75*E* − 01	−3.40*E* − 02
sANN_PID	3.78*E* − 01	2.43*E* − 01	2.02*E* − 01	7.59*E* − 01	−1.83*E* − 02	4.15*E* − 01	2.74*E* − 01	1.80*E* − 01	6.74*E* − 01	−3.70*E* − 02
hANN_MSE	3.63**E** − 01	2.23*E* − 01	1.71*E* − 01	7.61*E* − 01	6.56**E** − 02	3.93*E* − 01	2.43*E* − 01	1.56*E* − 01	6.59*E* − 01	8.02*E* − 02
hANN_tPI	3.64*E* − 01	2.24*E* − 01	1.67*E* − 01	7.62*E* − 01	5.93*E* − 02	3.83**E** − 01	2.36*E* − 01	1.55*E* − 01	6.54*E* − 01	1.05*E* − 01
hANN_PID	3.63**E** − 01	2.25*E* − 01	1.75*E* − 01	7.63**E** − 01	5.71*E* − 02	3.85*E* − 01	2.38*E* − 01	1.60*E* − 01	6.67*E* − 01	9.64*E* − 02
*Santa Ysabel Creek*										
3-4-1										
sANN_MSE	3.41*E* − 01	2.28*E* − 01	1.74*E* − 01	5.48*E* − 01	3.68*E* − 02	4.75*E* − 01	3.83*E* − 01	1.99*E* − 01	6.66*E* − 01	−2.88*E* − 01
sANN_tPI	3.50*E* − 01	2.35*E* − 01	1.61*E* − 01	5.34*E* − 01	7.74*E* − 03	4.36*E* − 01	3.52*E* − 01	1.85*E* − 01	6.93*E* − 01	−1.83*E* − 01
sANN_PID	3.45*E* − 01	2.30*E* − 01	1.67*E* − 01	5.43*E* − 01	2.75*E* − 02	4.75*E* − 01	3.85*E* − 01	2.01*E* − 01	6.64*E* − 01	−2.95*E* − 01
hANN_MSE	3.03*E* − 01	2.10*E* − 01	1.41*E* − 01	5.29*E* − 01	1.13*E* − 01	3.59*E* − 01	2.86*E* − 01	1.61**E** − 01	5.64*E* − 01	3.75*E* − 02
hANN_tPI	3.09*E* − 01	2.09*E* − 01	1.36**E** − 01	5.17*E* − 01	1.16*E* − 01	3.57*E* − 01	2.83*E* − 01	1.62*E* − 01	6.30*E* − 01	4.73*E* − 02
hANN_PID	2.97**E** − 01	2.08*E* − 01	1.44*E* − 01	5.36*E* − 01	1.20*E* − 01	3.38**E** − 01	2.84*E* − 01	1.65*E* − 01	5.58*E* − 01	4.39*E* − 02
3-6-1										
sANN_MSE	3.42*E* − 01	2.29*E* − 01	1.67*E* − 01	5.46*E* − 01	3.31*E* − 02	4.49*E* − 01	3.57*E* − 01	1.89*E* − 01	6.89*E* − 01	−2.00*E* − 01
sANN_tPI	3.39*E* − 01	2.24*E* − 01	1.88*E* − 01	5.55*E* − 01	5.23*E* − 02	4.50*E* − 01	3.48*E* − 01	2.18*E* − 01	6.96*E* − 01	−1.70*E* − 01
sANN_PID	3.30*E* − 01	2.22*E* − 01	1.73*E* − 01	5.60*E* − 01	6.21*E* − 02	4.34*E* − 01	3.45*E* − 01	2.01*E* − 01	6.99*E* − 01	−1.60*E* − 01
hANN_MSE	3.02*E* − 01	2.07*E* − 01	1.53*E* − 01	5.54*E* − 01	1.26*E* − 01	3.43*E* − 01	2.78*E* − 01	1.76*E* − 01	6.53*E* − 01	6.31*E* − 02
hANN_tPI	3.06*E* − 01	2.06**E** − 01	1.50*E* − 01	5.58*E* − 01	1.28*E* − 01	3.55*E* − 01	2.78*E* − 01	1.76*E* − 01	6.20*E* − 01	6.58*E* − 02
hANN_PID	3.04*E* − 01	2.08*E* − 01	1.59*E* − 01	5.56*E* − 01	1.22*E* − 01	3.55*E* − 01	2.76*E* − 01	1.82*E* − 01	6.39*E* − 01	7.16**E** − 02
3-8-1										
sANN_MSE	3.44*E* − 01	2.23*E* − 01	1.87*E* − 01	5.57*E* − 01	5.68*E* − 02	4.50*E* − 01	3.58*E* − 01	2.16*E* − 01	6.88*E* − 01	−2.04*E* − 01
sANN_tPI	3.30*E* − 01	2.24*E* − 01	1.89*E* − 01	5.56*E* − 01	5.42*E* − 02	4.19*E* − 01	3.30*E* − 01	2.21*E* − 01	7.12*E* − 01	−1.11*E* − 01
sANN_PID	3.27*E* − 01	2.22*E* − 01	1.90*E* − 01	5.60*E* − 01	6.32*E* − 02	4.22*E* − 01	3.27*E* − 01	2.23*E* − 01	7.14**E** − 01	−1.00*E* − 01
hANN_MSE	3.04*E* − 01	2.06**E** − 01	1.68*E* − 01	5.62*E* − 01	1.29*E* − 01	3.67*E* − 01	2.85*E* − 01	1.93*E* − 01	6.42*E* − 01	4.24*E* − 02
hANN_tPI	3.02*E* − 01	2.06**E** − 01	1.57*E* − 01	5.63**E** − 01	1.31**E** − 01	3.54*E* − 01	2.77**E** − 01	1.80*E* − 01	6.30*E* − 01	6.66*E* − 02
hANN_PID	3.07*E* − 01	2.06**E** − 01	1.55*E* − 01	5.54*E* − 01	1.29*E* − 01	3.82*E* − 01	2.91*E* − 01	1.81*E* − 01	6.36*E* − 01	1.99*E* − 02

**Table 3:** The medians of performance metrics for MLP architecture 6-*N*_hd_-1 on SPI forecast for sANN_TA and hANN_TA; TA shows the training error function, and the best models are marked with bold fonts.

	Calibration period					Validation period				
	MAE	MSE	dMSE	NS	PI	MAE	MSE	dMSE	NS	PI
*Leaf River*										
6-4-1										
sANN_MSE	5.76*E* − 01	5.25*E* − 01	2.71*E* − 01	4.78*E* − 01	−1.20*E* + 00	5.92*E* − 01	5.43*E* − 01	2.70*E* − 01	3.52*E* − 01	−1.06*E* + 00
sANN_tPI	5.91*E* − 01	5.53*E* − 01	2.92*E* − 01	4.50*E* − 01	−1.32*E* + 00	6.18*E* − 01	6.09*E* − 01	3.02*E* − 01	2.74*E* − 01	−1.31*E* + 00
sANN_PID	5.78*E* − 01	5.28*E* − 01	2.53*E* − 01	4.75*E* − 01	−1.22*E* + 00	6.20*E* − 01	5.94*E* − 01	2.40*E* − 01	2.91*E* − 01	−1.25*E* + 00
hANN_MSE	4.02*E* − 01	2.81*E* − 01	1.50*E* − 01	6.13*E* − 01	−1.79*E* − 01	4.46*E* − 01	3.24*E* − 01	1.53*E* − 01	4.32*E* − 01	−2.30*E* − 01
hANN_dMSE	2.81*E* + 00	8.90*E* + 00	1.10**E** − 01	−6.17*E* + 03	−3.63*E* + 01	3.19*E* + 00	1.10*E* + 01	1.15**E** − 01	−7.32*E* + 03	−4.08*E* + 01
hANN_tPI	3.91*E* − 01	2.53*E* − 01	1.48*E* − 01	6.09*E* − 01	−6.22*E* − 02	4.39*E* − 01	3.06*E* − 01	1.50*E* − 01	4.27*E* − 01	−1.59*E* − 01
hANN_PID	3.99*E* − 01	2.61*E* − 01	1.46*E* − 01	5.98*E* − 01	−9.73*E* − 02	4.45*E* − 01	3.08*E* − 01	1.51*E* − 01	3.89*E* − 01	−1.66*E* − 01
6-6-1										
sANN_MSE	5.66*E* − 01	5.12*E* − 01	2.31*E* − 01	4.91*E* − 01	−1.15*E* + 00	6.22*E* − 01	5.84*E* − 01	2.38*E* − 01	3.03*E* − 01	−1.22*E* + 00
sANN_tPI	5.90*E* − 01	5.51*E* − 01	2.81*E* − 01	4.52*E* − 01	−1.31*E* + 00	6.22*E* − 01	5.77*E* − 01	2.74*E* − 01	3.11*E* − 01	−1.19*E* + 00
sANN_PID	5.74*E* − 01	5.33*E* − 01	2.39*E* − 01	4.70*E* − 01	−1.24*E* + 00	6.27*E* − 01	5.99*E* − 01	2.33*E* − 01	2.85*E* − 01	−1.27*E* + 00
hANN_MSE	3.95*E* − 01	2.62*E* − 01	1.41*E* − 01	6.16**E** − 01	−1.01*E* − 01	4.21*E* − 01	2.74*E* − 01	1.54*E* − 01	4.36**E** − 01	−4.04*E* − 02
hANN_tPI	3.91*E* − 01	2.57*E* − 01	1.36*E* − 01	6.05*E* − 01	−7.68*E* − 02	4.19*E* − 01	2.70*E* − 01	1.40*E* − 01	4.24*E* − 01	−2.43*E* − 02
hANN_PID	3.86*E* − 01	2.55*E* − 01	1.47*E* − 01	6.04*E* − 01	−7.19*E* − 02	4.20*E* − 01	2.87*E* − 01	1.55*E* − 01	3.98*E* − 01	−8.81*E* − 02
6-8-1										
sANN_MSE	5.99*E* − 01	5.72*E* − 01	2.90*E* − 01	4.31*E* − 01	−1.40*E* + 00	6.40*E* − 01	6.21*E* − 01	3.06*E* − 01	2.59*E* − 01	−1.35*E* + 00
sANN_tPI	6.16*E* − 01	5.80*E* − 01	3.24*E* − 01	4.24*E* − 01	−1.43*E* + 00	6.27*E* − 01	6.35*E* − 01	3.22*E* − 01	2.43*E* − 01	−1.41*E* + 00
sANN_PID	6.17*E* − 01	5.87*E* − 01	3.60*E* − 01	4.16*E* − 01	−1.46*E* + 00	6.39*E* − 01	6.16*E* − 01	3.26*E* − 01	2.65*E* − 01	−1.33*E* + 00
hANN_MSE	3.91*E* − 01	2.56*E* − 01	1.51*E* − 01	5.07*E* − 01	−7.55*E* − 02	3.99**E** − 01	2.51*E* − 01	1.44*E* − 01	2.86*E* − 01	4.80**E** − 02
hANN_tPI	3.83**E** − 01	2.47**E** − 01	1.46*E* − 01	5.11*E* − 01	−3.68**E** − 02	4.30*E* − 01	2.90*E* − 01	1.49*E* − 01	2.57*E* − 01	−9.82*E* − 02
hANN_PID	3.86*E* − 01	2.47**E** − 01	1.55*E* − 01	5.11*E* − 01	−3.72*E* − 02	3.98*E* − 01	2.49**E** − 01	1.56*E* − 01	3.12*E* − 01	5.66*E* − 02
*Santa Ysabel Creek*										
6-4-1										
sANN_MSE	5.00*E* − 01	4.04*E* − 01	2.01*E* − 01	1.97*E* − 01	−7.10*E* − 01	7.08*E* − 01	7.81*E* − 01	2.15*E* − 01	3.18*E* − 01	−1.63*E* + 00
sANN_tPI	5.24*E* − 01	4.27*E* − 01	1.95*E* − 01	1.53*E* − 01	−8.05*E* − 01	7.09*E* − 01	7.75*E* − 01	2.06*E* − 01	3.23*E* − 01	−1.61*E* + 00
sANN_PID	5.28*E* − 01	4.51*E* − 01	2.19*E* − 01	1.04*E* − 01	−9.09*E* − 01	7.73*E* − 01	9.23*E* − 01	2.25*E* − 01	1.94*E* − 01	−2.10*E* + 00
hANN_MSE	3.50*E* − 01	2.42*E* − 01	1.24*E* − 01	2.01*E* − 01	−2.44*E* − 02	4.09*E* − 01	3.28*E* − 01	1.47*E* − 01	−7.21*E* − 02	−1.03*E* − 01
hANN_tPI	3.26*E* − 01	2.26*E* − 01	1.25*E* − 01	2.15*E* − 01	4.59*E* − 02	4.15*E* − 01	3.31*E* − 01	1.42*E* − 01	1.26*E* − 01	−1.13*E* − 01
hANN_PID	3.45*E* − 01	2.30*E* − 01	1.24*E* − 01	1.64*E* − 01	2.83*E* − 02	4.26*E* − 01	3.53*E* − 01	1.46*E* − 01	−1.38*E* − 01	−1.89*E* − 01
6-6-1										
sANN_MSE	5.11*E* − 01	4.22*E* − 01	1.96*E* − 01	1.63*E* − 01	−7.82*E* − 01	6.17*E* − 01	6.03*E* − 01	2.21*E* − 01	4.73*E* − 01	−1.03*E* + 00
sANN_tPI	5.43*E* − 01	4.67*E* − 01	3.03*E* − 01	7.31*E* − 02	−9.75*E* − 01	7.55*E* − 01	8.69*E* − 01	3.11*E* − 01	2.41*E* − 01	−1.93*E* + 00
sANN_PID	5.22*E* − 01	4.26*E* − 01	2.44*E* − 01	1.54*E* − 01	−8.01*E* − 01	6.86*E* − 01	7.11*E* − 01	2.57*E* − 01	3.79*E* − 01	−1.39*E* + 00
hANN_MSE	3.42*E* − 01	2.22*E* − 01	1.26*E* − 01	3.35**E** − 01	6.10*E* − 02	4.08*E* − 01	3.31*E* − 01	1.51*E* − 01	3.30*E* − 01	−1.13*E* − 01
hANN_tPI	3.03**E** − 01	2.21*E* − 01	1.17**E** − 01	3.18*E* − 01	6.40*E* − 02	3.58**E** − 01	2.94**E** − 01	1.41**E** − 01	2.65*E* − 01	1.06**E** − 02
hANN_PID	3.36*E* − 01	2.19*E* − 01	1.18*E* − 01	3.06*E* − 01	7.34*E* − 02	4.01*E* − 01	3.07*E* − 01	1.43*E* − 01	2.84*E* − 01	−3.36*E* − 02
6-8-1										
sANN_MSE	5.15*E* − 01	4.38*E* − 01	2.78*E* − 01	1.32*E* − 01	−8.50*E* − 01	6.17*E* − 01	5.94*E* − 01	2.92*E* − 01	4.81**E** − 01	−9.99*E* − 01
sANN_tPI	5.24*E* − 01	4.45*E* − 01	2.72*E* − 01	1.17*E* − 01	−8.81*E* − 01	6.21*E* − 01	6.44*E* − 01	2.91*E* − 01	4.38*E* − 01	−1.17*E* + 00
sANN_PID	5.47*E* − 01	4.93*E* − 01	2.72*E* − 01	2.24*E* − 02	−1.08*E* + 00	6.73*E* − 01	7.15*E* − 01	2.84*E* − 01	3.76*E* − 01	−1.40*E* + 00
hANN_MSE	3.36*E* − 01	2.18**E** − 01	1.40*E* − 01	2.67*E* − 01	7.84**E** − 02	4.23*E* − 01	3.33*E* − 01	1.61*E* − 01	3.22*E* − 01	−1.20*E* − 01
hANN_tPI	3.33*E* − 01	2.25*E* − 01	1.37*E* − 01	2.74*E* − 01	5.07*E* − 02	4.04*E* − 01	3.28*E* − 01	1.53*E* − 01	3.65*E* − 01	−1.04*E* − 01
hANN_PID	3.31*E* − 01	2.31*E* − 01	1.41*E* − 01	2.50*E* − 01	2.30*E* − 02	3.75*E* − 01	3.11*E* − 01	1.68*E* − 01	2.03*E* − 01	−4.73*E* − 02

**Table 4:** The medians of performance metrics for MLP architecture 9-*N*_hd_-1 on SPI forecast for sANN_TA and hANN_TA; TA shows the training error function, and the best models are marked with bold fonts.

	Calibration period					Validation period				
	MAE	MSE	dMSE	NS	PI	MAE	MSE	dMSE	NS	PI
*Leaf River*										
9-4-1										
sANN_MSE	3.77*E* − 01	2.35*E* − 01	1.71*E* − 01	7.51*E* − 01	7.42*E* − 01	4.54*E* − 01	3.38*E* − 01	1.89*E* − 01	6.05*E* − 01	7.31*E* − 01
sANN_tPI	3.79*E* − 01	2.36*E* − 01	1.98*E* − 01	7.50*E* − 01	7.41*E* − 01	4.60*E* − 01	3.44*E* − 01	2.16*E* − 01	5.98*E* − 01	7.26*E* − 01
sANN_PID	3.86*E* − 01	2.45*E* − 01	1.52*E* − 01	7.41*E* − 01	7.32*E* − 01	4.62*E* − 01	3.50*E* − 01	1.75*E* − 01	5.91*E* − 01	7.22*E* − 01
hANN_MSE	3.46*E* − 01	2.01*E* − 01	1.54*E* − 01	7.61*E* − 01	7.80*E* − 01	4.09*E* − 01	2.73*E* − 01	1.62*E* − 01	5.91*E* − 01	7.83*E* − 01
hANN_tPI	3.44*E* − 01	2.02*E* − 01	1.53*E* − 01	7.66*E* − 01	3.86*E* − 02	4.16*E* − 01	2.83*E* − 01	1.62*E* − 01	5.79*E* − 01	7.75*E* − 01
hANN_PID	3.47*E* − 01	2.01*E* − 01	1.36**E** − 01	7.57*E* − 01	7.79*E* − 01	4.08*E* − 01	2.74*E* − 01	1.57*E* − 01	5.75*E* − 01	7.82*E* − 01
9-6-1										
sANN_MSE	3.75*E* − 01	2.29*E* − 01	2.00*E* − 01	7.57*E* − 01	7.49*E* − 01	4.44*E* − 01	3.23*E* − 01	2.14*E* − 01	6.23*E* − 01	7.43*E* − 01
sANN_tPI	3.72*E* − 01	2.28*E* − 01	1.88*E* − 01	7.59*E* − 01	7.50*E* − 01	4.45*E* − 01	3.21*E* − 01	2.08*E* − 01	6.25*E* − 01	7.45*E* − 01
sANN_PID	3.82*E* − 01	2.45*E* − 01	1.63*E* − 01	7.41*E* − 01	7.32*E* − 01	4.69*E* − 01	3.63*E* − 01	1.86*E* − 01	5.76*E* − 01	7.11*E* − 01
hANN_MSE	3.42**E** − 01	1.98*E* − 01	1.57*E* − 01	7.68*E* − 01	7.84*E* − 01	4.08*E* − 01	2.79*E* − 01	1.69*E* − 01	5.88*E* − 01	7.79*E* − 01
hANN_tPI	3.44*E* − 01	1.99*E* − 01	1.59*E* − 01	7.70*E* − 01	7.82*E* − 01	4.03**E** − 01	2.68**E** − 01	1.74*E* − 01	6.03*E* − 01	7.87**E** − 01
hANN_PID	3.46*E* − 01	2.02*E* − 01	1.44*E* − 01	7.53*E* − 01	7.79*E* − 01	4.10*E* − 01	2.73*E* − 01	1.50**E** − 01	5.87*E* − 01	7.83*E* − 01
9-8-1										
sANN_MSE	3.72*E* − 01	2.29*E* − 01	1.91*E* − 01	7.57*E* − 01	7.49*E* − 01	4.41*E* − 01	3.19*E* − 01	2.15*E* − 01	6.27**E** − 01	7.46*E* − 01
sANN_tPI	3.79*E* − 01	2.38*E* − 01	1.96*E* − 01	7.48*E* − 01	7.39*E* − 01	4.54*E* − 01	3.36*E* − 01	2.18*E* − 01	6.07*E* − 01	7.33*E* − 01
sANN_PID	3.78*E* − 01	2.44*E* − 01	1.65*E* − 01	7.42*E* − 01	7.33*E* − 01	4.66*E* − 01	3.50*E* − 01	1.85*E* − 01	5.91*E* − 01	7.22*E* − 01
hANN_MSE	3.45*E* − 01	1.96**E** − 01	1.57*E* − 01	7.75**E** − 01	7.85**E** − 01	4.04*E* − 01	2.69*E* − 01	1.71*E* − 01	6.07*E* − 01	7.86*E* − 01
hANN_tPI	3.45*E* − 01	1.97*E* − 01	1.64*E* − 01	7.75**E** − 01	7.84*E* − 01	4.08*E* − 01	2.74*E* − 01	1.71*E* − 01	5.94*E* − 01	7.82*E* − 01
hANN_PID	3.44*E* − 01	1.98*E* − 01	1.49*E* − 01	7.66*E* − 01	7.83*E* − 01	4.07*E* − 01	2.77*E* − 01	1.63*E* − 01	6.04*E* − 01	7.80*E* − 01
*Santa Ysabel Creek*										
9-4-1										
sANN_MSE	3.97*E* − 01	2.88*E* − 01	2.27*E* − 01	5.56*E* − 01	7.64*E* − 01	5.37*E* − 01	4.50*E* − 01	2.12*E* − 01	6.25*E* − 01	6.45*E* − 01
sANN_tPI	4.05*E* − 01	2.90*E* − 01	2.33*E* − 01	5.53*E* − 01	7.62*E* − 01	5.59*E* − 01	4.77*E* − 01	2.20*E* − 01	6.02*E* − 01	6.23*E* − 01
sANN_PID	4.17*E* − 01	3.07*E* − 01	1.87*E* − 01	5.27*E* − 01	7.48*E* − 01	6.39*E* − 01	5.99*E* − 01	1.74*E* − 01	5.01*E* − 01	5.27*E* − 01
hANN_MSE	3.57*E* − 01	2.52*E* − 01	1.98*E* − 01	5.75*E* − 01	7.93*E* − 01	4.61*E* − 01	3.49*E* − 01	1.81*E* − 01	5.07*E* − 01	7.25*E* − 01
hANN_tPI	3.56*E* − 01	2.48*E* − 01	1.90*E* − 01	5.77*E* − 01	7.96*E* − 01	4.52*E* − 01	3.36*E* − 01	1.75*E* − 01	5.50*E* − 01	7.34*E* − 01
hANN_PID	3.67*E* − 01	2.62*E* − 01	1.76*E* − 01	5.51*E* − 01	7.85*E* − 01	5.04*E* − 01	3.97*E* − 01	1.65*E* − 01	4.70*E* − 01	6.87*E* − 01
9-6-1										
sANN_MSE	4.06*E* − 01	2.88*E* − 01	2.43*E* − 01	5.56*E* − 01	7.64*E* − 01	5.42*E* − 01	4.55*E* − 01	2.20*E* − 01	6.20*E* − 01	6.41*E* − 01
sANN_tPI	3.98*E* − 01	2.85*E* − 01	2.59*E* − 01	5.61*E* − 01	7.66*E* − 01	5.43*E* − 01	4.48*E* − 01	2.44*E* − 01	6.26*E* − 01	6.46*E* − 01
sANN_PID	4.22*E* − 01	3.07*E* − 01	1.95*E* − 01	5.26*E* − 01	7.48*E* − 01	6.36*E* − 01	6.02*E* − 01	1.81*E* − 01	4.98*E* − 01	5.25*E* − 01
hANN_MSE	3.56*E* − 01	2.48*E* − 01	1.96*E* − 01	5.78**E** − 01	7.96*E* − 01	4.73*E* − 01	3.59*E* − 01	1.83*E* − 01	5.77*E* − 01	7.16*E* − 01
hANN_tPI	3.58*E* − 01	2.49*E* − 01	2.02*E* − 01	5.74*E* − 01	7.96*E* − 01	4.46*E* − 01	3.34*E* − 01	1.86*E* − 01	5.72*E* − 01	7.36*E* − 01
hANN_PID	3.75*E* − 01	2.60*E* − 01	1.75**E** − 01	5.23*E* − 01	7.87*E* − 01	5.35*E* − 01	4.34*E* − 01	1.61**E** − 01	4.41*E* − 01	6.57*E* − 01
9-8-1										
sANN_MSE	4.08*E* − 01	2.91*E* − 01	2.68*E* − 01	5.52*E* − 01	7.61*E* − 01	5.16*E* − 01	4.13*E* − 01	2.41*E* − 01	6.56**E** − 01	6.74*E* − 01
sANN_tPI	4.04*E* − 01	2.90*E* − 01	2.49*E* − 01	5.54*E* − 01	7.62*E* − 01	5.38*E* − 01	4.38*E* − 01	2.39*E* − 01	6.34*E* − 01	6.54*E* − 01
sANN_PID	4.24*E* − 01	3.13*E* − 01	1.99*E* − 01	5.18*E* − 01	7.43*E* − 01	6.34*E* − 01	6.00*E* − 01	1.85*E* − 01	4.99*E* − 01	5.26*E* − 01
hANN_MSE	3.54**E** − 01	2.46**E** − 01	1.90*E* − 01	5.74*E* − 01	7.98**E** − 01	4.41**E** − 01	3.55*E* − 01	1.86*E* − 01	5.47*E* − 01	7.20*E* − 01
hANN_tPI	3.56*E* − 01	2.49*E* − 01	2.01*E* − 01	5.78**E** − 01	7.95*E* − 01	4.42*E* − 01	3.31**E** − 01	1.94*E* − 01	5.28*E* − 01	7.38**E** − 01
hANN_PID	3.66*E* − 01	2.61*E* − 01	1.75**E** − 01	5.44*E* − 01	7.86*E* − 01	5.34*E* − 01	4.34*E* − 01	1.61**E** − 01	4.76*E* − 01	6.57*E* − 01

**Table 5:** The values of PI2 on architectures 9-*N*_hd_-1 for SPI forecast with hANN.

	Calibration period			Validation period		
	9-4-1	9-6-1	9-8-1	9-4-1	9-6-1	9-8-1
*Leaf River *						
9-4-1	0.00*E* + 00	−1.52*E* − 02	−2.91*E* − 02	0.00*E* + 00	−6.31*E* − 03	−3.64*E* − 02
9-6-1	1.50*E* − 02	0.00*E* + 00	−1.37*E* − 02	6.27*E* − 03	0.00*E* + 00	−2.99*E* − 02
9-8-1	2.83*E* − 02	1.35*E* − 02	0.00*E* + 00	3.51*E* − 02	2.90*E* − 02	0.00*E* + 00
*Santa Ysabel *						
9-4-1	0.00*E* + 00	−6.92*E* − 03	−4.77*E* − 03	0.00*E* + 00	2.13*E* − 02	−1.83*E* − 03
9-6-1	6.88*E* − 03	0.00*E* + 00	2.13*E* − 03	−2.18*E* − 02	0.00*E* + 00	−2.36*E* − 02
9-8-1	4.75*E* − 03	−2.14*E* − 03	0.00*E* + 00	1.82*E* − 03	2.31*E* − 02	0.00*E* + 00

**Table 6:** The values of PI2 on training functions for SPI forecast with hANN.

	Calibration period			Validation period		
	MSE	tPI	PID	MSE	tPI	PID
*Leaf River *						
MSE	0.00*E* + 00	−2.94*E* − 03	1.64*E* − 02	0.00*E* + 00	−5.25*E* − 03	2.44*E* − 02
tPI	2.94*E* − 03	0.00*E* + 00	1.93*E* − 02	5.22*E* − 03	0.00*E* + 00	2.95*E* − 02
PID	−1.67*E* − 02	−1.97*E* − 02	0.00*E* + 00	−2.50*E* − 02	−3.04*E* − 02	0.00*E* + 00
*Santa Ysabel *						
MSE	0.00*E* + 00	−1.07*E* − 03	5.61*E* − 02	0.00*E* + 00	1.51*E* − 03	2.07*E* − 01
tPI	1.06*E* − 03	0.00*E* + 00	5.71*E* − 02	−1.51*E* − 03	0.00*E* + 00	2.06*E* − 01
PID	−5.94*E* − 02	−6.06*E* − 02	0.00*E* + 00	−2.61*E* − 01	−2.59*E* − 01	0.00*E* + 00

**Table 7:** The medians of performance metrics for MLP architecture 3-*N*_hd_-1 on SPEI forecast for sANN_TA and hANN_TA; TA shows the training error function, and the best models are marked with bold fonts.

	Calibration period					Validation period				
	MAE	MSE	dMSE	NS	PI	MAE	MSE	dMSE	NS	PI
*Leaf River*										
3-4-1										
sANN_MSE	3.76*E* − 01	2.36*E* − 01	1.63*E* − 01	7.62*E* − 01	−6.99*E* − 02	4.43*E* − 01	3.10*E* − 01	1.72*E* − 01	6.43*E* − 01	−1.28*E* − 01
sANN_tPI	3.80*E* − 01	2.46*E* − 01	1.71*E* − 01	7.52*E* − 01	−1.15*E* − 01	4.47*E* − 01	3.13*E* − 01	1.74*E* − 01	6.40*E* − 01	−1.37*E* − 01
sANN_PID	3.77*E* − 01	2.35*E* − 01	1.62*E* − 01	7.64*E* − 01	−6.34*E* − 02	4.46*E* − 01	3.12*E* − 01	1.76*E* − 01	6.41*E* − 01	−1.35*E* − 01
hANN_MSE	3.53*E* − 01	2.12*E* − 01	1.44*E* − 01	7.60*E* − 01	3.98*E* − 02	3.98*E* − 01	2.53*E* − 01	1.54*E* − 01	6.13*E* − 01	7.90*E* − 02
hANN_tPI	3.54*E* − 01	2.13*E* − 01	1.46*E* − 01	7.51*E* − 01	3.62*E* − 02	4.05*E* − 01	2.58*E* − 01	1.53*E* − 01	6.02*E* − 01	6.09*E* − 02
hANN_PID	3.51*E* − 01	2.10*E* − 01	1.43**E** − 01	7.48*E* − 01	5.07*E* − 02	4.05*E* − 01	2.61*E* − 01	1.52**E** − 01	5.91*E* − 01	5.05*E* − 02
3-6-1										
sANN_MSE	3.79*E* − 01	2.36*E* − 01	1.79*E* − 01	7.62*E* − 01	−7.07*E* − 02	4.37*E* − 01	3.00*E* − 01	1.86*E* − 01	6.55*E* − 01	−9.08*E* − 02
sANN_tPI	3.69*E* − 01	2.27*E* − 01	1.78*E* − 01	7.72*E* − 01	−2.66*E* − 02	4.30*E* − 01	2.91*E* − 01	1.83*E* − 01	6.65*E* − 01	−5.78*E* − 02
sANN_PID	3.70*E* − 01	2.32*E* − 01	1.68*E* − 01	7.67*E* − 01	−4.87*E* − 02	4.33*E* − 01	2.94*E* − 01	1.71*E* − 01	6.62*E* − 01	−6.89*E* − 02
hANN_MSE	3.52*E* − 01	2.08*E* − 01	1.47*E* − 01	7.64*E* − 01	5.87*E* − 02	3.98*E* − 01	2.52*E* − 01	1.55*E* − 01	6.46*E* − 01	8.21*E* − 02
hANN_tPI	3.51*E* − 01	2.08*E* − 01	1.53*E* − 01	7.62*E* − 01	5.94*E* − 02	3.97*E* − 01	2.53*E* − 01	1.60*E* − 01	6.34*E* − 01	8.07*E* − 02
hANN_PID	3.55*E* − 01	2.09*E* − 01	1.52*E* − 01	7.63*E* − 01	5.41*E* − 02	3.95**E** − 01	2.53*E* − 01	1.60*E* − 01	6.30*E* − 01	8.12*E* − 02
3-8-1										
sANN_MSE	3.71*E* − 01	2.24*E* − 01	2.02*E* − 01	7.75**E** − 01	−1.34*E* − 02	4.20*E* − 01	2.78*E* − 01	2.08*E* − 01	6.80**E** − 01	−1.18*E* − 02
sANN_tPI	3.72*E* − 01	2.28*E* − 01	1.75*E* − 01	7.71*E* − 01	−3.12*E* − 02	4.24*E* − 01	2.86*E* − 01	1.83*E* − 01	6.71*E* − 01	−3.81*E* − 02
sANN_PID	3.71*E* − 01	2.27*E* − 01	1.99*E* − 01	7.72*E* − 01	−2.74*E* − 02	4.22*E* − 01	2.86*E* − 01	2.01*E* − 01	6.71*E* − 01	−3.94*E* − 02
hANN_MSE	3.51*E* − 01	2.09*E* − 01	1.57*E* − 01	7.69*E* − 01	5.25*E* − 02	3.95**E** − 01	2.48*E* − 01	1.66*E* − 01	6.59*E* − 01	9.83*E* − 02
hANN_tPI	3.50**E** − 01	2.08*E* − 01	1.64*E* − 01	7.73*E* − 01	5.96*E* − 02	3.98*E* − 01	2.52*E* − 01	1.68*E* − 01	6.55*E* − 01	8.28*E* − 02
hANN_PID	3.50**E** − 01	2.07**E** − 01	1.62*E* − 01	7.68*E* − 01	6.29**E** − 02	3.90*E* − 01	2.46**E** − 01	1.73*E* − 01	6.43*E* − 01	1.04**E** − 01
*Santa Ysabel Creek*										
3-4-1										
sANN_MSE	3.97*E* − 01	2.93*E* − 01	2.25*E* − 01	5.42*E* − 01	3.03*E* − 02	4.64*E* − 01	3.55*E* − 01	2.02*E* − 01	6.94*E* − 01	−1.87*E* − 01
sANN_tPI	3.88*E* − 01	2.93*E* − 01	2.13*E* − 01	5.42*E* − 01	3.08*E* − 02	4.66*E* − 01	3.62*E* − 01	1.95*E* − 01	6.88*E* − 01	−2.11*E* − 01
sANN_PID	4.04*E* − 01	2.93*E* − 01	2.16*E* − 01	5.42*E* − 01	3.01*E* − 02	5.18*E* − 01	4.03*E* − 01	1.99*E* − 01	6.53*E* − 01	−3.48*E* − 01
hANN_MSE	3.50*E* − 01	2.66*E* − 01	1.89*E* − 01	5.45*E* − 01	1.22*E* − 01	3.62*E* − 01	2.86*E* − 01	1.71**E** − 01	6.26*E* − 01	4.41*E* − 02
hANN_tPI	3.44*E* − 01	2.63**E** − 01	1.95*E* − 01	5.28*E* − 01	1.30**E** − 01	3.71*E* − 01	2.81*E* − 01	1.79*E* − 01	6.39*E* − 01	5.93*E* − 02
hANN_PID	3.54*E* − 01	2.65*E* − 01	1.80**E** − 01	5.39*E* − 01	1.25*E* − 01	3.76*E* − 01	2.84*E* − 01	1.65*E* − 01	5.70*E* − 01	5.03*E* − 02
3-6-1										
sANN_MSE	3.95*E* − 01	2.84*E* − 01	2.29*E* − 01	5.56*E* − 01	6.01*E* − 02	4.90*E* − 01	3.82*E* − 01	2.06*E* − 01	6.71*E* − 01	−2.77*E* − 01
sANN_tPI	3.83*E* − 01	2.87*E* − 01	2.31*E* − 01	5.52*E* − 01	5.14*E* − 02	4.65*E* − 01	3.48*E* − 01	2.08*E* − 01	7.01*E* − 01	−1.63*E* − 01
sANN_PID	3.89*E* − 01	2.85*E* − 01	2.20*E* − 01	5.55*E* − 01	5.65*E* − 02	4.63*E* − 01	3.48*E* − 01	1.91*E* − 01	7.00*E* − 01	−1.64*E* − 01
hANN_MSE	3.47*E* − 01	2.66*E* − 01	1.93*E* − 01	5.59*E* − 01	1.19*E* − 01	3.80*E* − 01	2.89*E* − 01	1.77*E* − 01	6.51*E* − 01	3.15*E* − 02
hANN_tPI	3.51*E* − 01	2.64*E* − 01	1.87*E* − 01	5.50*E* − 01	1.27*E* − 01	3.62*E* − 01	2.77**E** − 01	1.76*E* − 01	6.52*E* − 01	7.21**E** − 02
hANN_PID	3.52*E* − 01	2.64*E* − 01	1.93*E* − 01	5.52*E* − 01	1.27*E* − 01	3.62*E* − 01	2.78*E* − 01	1.76*E* − 01	6.59*E* − 01	7.05*E* − 02
3-8-1										
sANN_MSE	3.80*E* − 01	2.83*E* − 01	2.39*E* − 01	5.58*E* − 01	6.39*E* − 02	4.51*E* − 01	3.37*E* − 01	2.17*E* − 01	7.10*E* − 01	−1.28*E* − 01
sANN_tPI	3.92*E* − 01	2.85*E* − 01	2.26*E* − 01	5.54*E* − 01	5.63*E* − 02	4.77*E* − 01	3.68*E* − 01	2.09*E* − 01	6.83*E* − 01	−2.30*E* − 01
sANN_PID	3.82*E* − 01	2.83*E* − 01	2.39*E* − 01	5.58*E* − 01	6.37*E* − 02	4.32*E* − 01	3.30*E* − 01	2.20*E* − 01	7.16**E** − 01	−1.04*E* − 01
hANN_MSE	3.49*E* − 01	2.64*E* − 01	2.00*E* − 01	5.60*E* − 01	1.26*E* − 01	3.91*E* − 01	2.90*E* − 01	1.80*E* − 01	6.51*E* − 01	3.04*E* − 02
hANN_tPI	3.46*E* − 01	2.64*E* − 01	2.01*E* − 01	5.57*E* − 01	1.27*E* − 01	3.72*E* − 01	2.82*E* − 01	1.83*E* − 01	6.75*E* − 01	5.50*E* − 02
hANN_PID	3.39**E** − 01	2.63**E** − 01	2.08*E* − 01	5.63**E** − 01	1.29*E* − 01	3.58**E** − 01	2.81*E* − 01	1.90*E* − 01	6.53*E* − 01	6.12*E* − 02

**Table 8:** The medians of performance metrics for MLP architecture 6-*N*_hd_-1 on SPEI forecast for sANN_TA and hANN_TA; TA shows the training error function, and the best models are marked with bold fonts.

	Calibration period					Validation period				
	MAE	MSE	dMSE	NS	PI	MAE	MSE	dMSE	NS	PI
*Leaf River*										
6-4-1										
ANN_MSE	5.99*E* − 01	5.66*E* − 01	2.41*E* − 01	4.31*E* − 01	−1.56*E* + 00	6.40*E* − 01	6.13*E* − 01	2.61*E* − 01	2.95*E* − 01	−1.23*E* + 00
ANN_tPI	5.76*E* − 01	5.32*E* − 01	2.34*E* − 01	4.65*E* − 01	−1.41*E* + 00	6.34*E* − 01	6.14*E* − 01	2.49*E* − 01	2.94*E* − 01	−1.23*E* + 00
ANN_PID	5.83*E* − 01	5.24*E* − 01	2.24*E* − 01	4.73*E* − 01	−1.37*E* + 00	6.22*E* − 01	5.81*E* − 01	2.36*E* − 01	3.32*E* − 01	−1.11*E* + 00
hANN_MSE	3.78*E* − 01	2.41*E* − 01	1.38*E* − 01	5.47*E* − 01	−9.35*E* − 02	4.47*E* − 01	3.19*E* − 01	1.51*E* − 01	3.94*E* − 01	−1.59*E* − 01
hANN_tPI	3.92*E* − 01	2.56*E* − 01	1.26**E** − 01	5.75*E* − 01	−1.60*E* − 01	4.42*E* − 01	3.08*E* − 01	1.44**E** − 01	3.56*E* − 01	−1.19*E* − 01
hANN_PID	3.84*E* − 01	2.52*E* − 01	1.32*E* − 01	5.56*E* − 01	−1.41*E* − 01	4.45*E* − 01	3.16*E* − 01	1.51*E* − 01	3.51*E* − 01	−1.49*E* − 01
6-6-1										
ANN_MSE	5.77*E* − 01	5.12*E* − 01	2.28*E* − 01	4.85*E* − 01	−1.32*E* + 00	6.45*E* − 01	6.44*E* − 01	2.29*E* − 01	2.59*E* − 01	−1.34*E* + 00
ANN_tPI	5.82*E* − 01	5.21*E* − 01	2.68*E* − 01	4.76*E* − 01	−1.36*E* + 00	6.27*E* − 01	5.96*E* − 01	2.94*E* − 01	3.14*E* − 01	−1.17*E* + 00
ANN_PID	6.06*E* − 01	5.68*E* − 01	3.26*E* − 01	4.29*E* − 01	−1.57*E* + 00	6.56*E* − 01	6.61*E* − 01	3.58*E* − 01	2.39*E* − 01	−1.40*E* + 00
hANN_MSE	3.81*E* − 01	2.43*E* − 01	1.26**E** − 01	5.90*E* − 01	−1.00*E* − 01	4.36*E* − 01	3.05*E* − 01	1.45*E* − 01	3.82*E* − 01	−1.09*E* − 01
hANN_tPI	3.90*E* − 01	2.42*E* − 01	1.48*E* − 01	6.16*E* − 01	−9.60*E* − 02	4.17*E* − 01	2.78*E* − 01	1.68*E* − 01	3.80*E* − 01	−9.50*E* − 03
hANN_PID	3.87*E* − 01	2.49*E* − 01	1.39*E* − 01	5.49*E* − 01	−1.26*E* − 01	4.20*E* − 01	2.86*E* − 01	1.56*E* − 01	2.55*E* − 01	−4.12*E* − 02
6-8-1										
ANN_MSE	6.13*E* − 01	5.89*E* − 01	3.39*E* − 01	4.07*E* − 01	−1.67*E* + 00	6.79*E* − 01	6.81*E* − 01	3.51*E* − 01	2.16*E* − 01	−1.48*E* + 00
ANN_tPI	6.01*E* − 01	5.56*E* − 01	3.29*E* − 01	4.40*E* − 01	−1.52*E* + 00	6.37*E* − 01	6.17*E* − 01	3.31*E* − 01	2.90*E* − 01	−1.24*E* + 00
ANN_PID	6.35*E* − 01	6.00*E* − 01	2.93*E* − 01	3.96*E* − 01	−1.72*E* + 00	6.56*E* − 01	6.88*E* − 01	3.24*E* − 01	2.08*E* − 01	−1.50*E* + 00
hANN_MSE	3.70**E** − 01	2.35*E* − 01	1.38*E* − 01	5.80*E* − 01	−6.30*E* − 02	4.23*E* − 01	2.79*E* − 01	1.56*E* − 01	3.97*E* − 01	−1.53*E* − 02
hANN_tPI	3.70**E** − 01	2.29**E** − 01	1.35*E* − 01	6.12*E* − 01	−3.49**E** − 02	4.16**E** − 01	2.75**E** − 01	1.54*E* − 01	3.64*E* − 01	6.22**E** − 04
hANN_PID	3.78*E* − 01	2.41*E* − 01	1.32*E* − 01	6.34**E** − 01	−8.97*E* − 02	4.23*E* − 01	2.81*E* − 01	1.49*E* − 01	4.34**E** − 01	−2.32*E* − 02
*Santa Ysabel Creek*										
6-4-1										
ANN_MSE	5.62*E* − 01	5.01*E* − 01	2.22*E* − 01	2.18*E* − 01	−6.57*E* − 01	6.73*E* − 01	6.78*E* − 01	1.92*E* − 01	4.16*E* − 01	−1.27*E* + 00
ANN_tPI	5.64*E* − 01	5.11*E* − 01	2.89*E* − 01	2.03*E* − 01	−6.88*E* − 01	6.83*E* − 01	7.80*E* − 01	2.65*E* − 01	3.28*E* − 01	−1.61*E* + 00
ANN_PID	5.56*E* − 01	4.86*E* − 01	2.18*E* − 01	2.42*E* − 01	−6.05*E* − 01	7.12*E* − 01	7.41*E* − 01	2.00*E* − 01	3.62*E* − 01	−1.48*E* + 00
hANN_MSE	3.78*E* − 01	2.95*E* − 01	1.75*E* − 01	2.71*E* − 01	2.47*E* − 02	4.33*E* − 01	3.43*E* − 01	1.60*E* − 01	2.43*E* − 01	−1.46*E* − 01
hANN_tPI	4.12*E* − 01	3.14*E* − 01	1.61**E** − 01	2.24*E* − 01	−3.79*E* − 02	4.31*E* − 01	3.52*E* − 01	1.46**E** − 01	2.79*E* − 02	−1.79*E* − 01
hANN_PID	4.08*E* − 01	3.05*E* − 01	1.73*E* − 01	2.33*E* − 01	−6.59*E* − 03	4.36*E* − 01	3.38*E* − 01	1.56*E* − 01	3.06*E* − 01	−1.30*E* − 01
6-6-1										
ANN_MSE	5.31*E* − 01	4.85*E* − 01	2.89*E* − 01	2.43*E* − 01	−6.04*E* − 01	5.88*E* − 01	5.57*E* − 01	2.60*E* − 01	5.20*E* − 01	−8.65*E* − 01
ANN_tPI	5.44*E* − 01	4.87*E* − 01	2.48*E* − 01	2.40*E* − 01	−6.11*E* − 01	5.88*E* − 01	5.52*E* − 01	2.36*E* − 01	5.25**E** − 01	−8.45*E* − 01
ANN_PID	5.42*E* − 01	5.01*E* − 01	2.58*E* − 01	2.18*E* − 01	−6.56*E* − 01	6.57*E* − 01	6.37*E* − 01	2.34*E* − 01	4.51*E* − 01	−1.13*E* + 00
hANN_MSE	3.67**E** − 01	2.78*E* − 01	1.66*E* − 01	3.97**E** − 01	8.27*E* − 02	3.80**E** − 01	3.18*E* − 01	1.54*E* − 01	4.69*E* − 01	−6.28*E* − 02
hANN_tPI	3.81*E* − 01	2.80*E* − 01	1.73*E* − 01	3.89*E* − 01	7.60*E* − 02	4.14*E* − 01	3.28*E* − 01	1.59*E* − 01	4.93*E* − 01	−9.72*E* − 02
hANN_PID	3.71*E* − 01	2.76**E** − 01	1.66*E* − 01	3.87*E* − 01	8.82**E** − 02	4.18*E* − 01	3.15*E* − 01	1.54*E* − 01	4.61*E* − 01	−5.42*E* − 02
6-8-1										
ANN_MSE	5.97*E* − 01	5.78*E* − 01	2.60*E* − 01	9.81*E* − 02	−9.10*E* − 01	7.22*E* − 01	8.26*E* − 01	2.35*E* − 01	2.88*E* − 01	−1.77*E* + 00
ANN_tPI	5.60*E* − 01	5.37*E* − 01	3.04*E* − 01	1.62*E* − 01	−7.75*E* − 01	6.42*E* − 01	6.24*E* − 01	2.78*E* − 01	4.62*E* − 01	−1.09*E* + 00
ANN_PID	5.52*E* − 01	5.21*E* − 01	2.79*E* − 01	1.86*E* − 01	−7.24*E* − 01	6.86*E* − 01	7.00*E* − 01	2.39*E* − 01	3.97*E* − 01	−1.34*E* + 00
hANN_MSE	3.87*E* − 01	2.92*E* − 01	1.64*E* − 01	3.49*E* − 01	3.36*E* − 02	4.49*E* − 01	3.43*E* − 01	1.54*E* − 01	3.95*E* − 01	−1.47*E* − 01
hANN_tPI	3.75*E* − 01	2.85*E* − 01	1.73*E* − 01	3.69*E* − 01	5.67*E* − 02	4.18*E* − 01	3.27*E* − 01	1.54*E* − 01	4.79*E* − 01	−9.51*E* − 02
hANN_PID	3.79*E* − 01	2.94*E* − 01	1.72*E* − 01	3.49*E* − 01	2.75*E* − 02	3.89*E* − 01	3.12**E** − 01	1.58*E* − 01	4.21*E* − 01	−4.46**E** − 02

**Table 9:** The medians of performance metrics for MLP architecture 9-*N*_hd_-1 on SPEI forecast for sANN_TA and hANN_TA; TA shows the training error function, and the best models are marked with bold fonts.

	Calibration period					Validation period				
	MAE	MSE	dMSE	NS	PI	MAE	MSE	dMSE	NS	PI
*Leaf River*										
9-4-1										
sANN_MSE	3.75*E* − 01	2.36*E* − 01	1.97*E* − 01	7.50*E* − 01	7.41*E* − 01	4.53*E* − 01	3.34*E* − 01	2.07*E* − 01	6.09*E* − 01	7.35*E* − 01
sANN_tPI	3.77*E* − 01	2.32*E* − 01	1.84*E* − 01	7.54*E* − 01	7.45*E* − 01	4.52*E* − 01	3.46*E* − 01	2.11*E* − 01	5.96*E* − 01	7.26*E* − 01
sANN_PID	3.81*E* − 01	2.40*E* − 01	1.63*E* − 01	7.46*E* − 01	7.37*E* − 01	4.55*E* − 01	3.43*E* − 01	1.76*E* − 01	5.99*E* − 01	7.28*E* − 01
hANN_MSE	3.47*E* − 01	2.00*E* − 01	1.54*E* − 01	7.63*E* − 01	7.81*E* − 01	4.04*E* − 01	2.66*E* − 01	1.57*E* − 01	5.91*E* − 01	7.89*E* − 01
hANN_tPI	3.45*E* − 01	2.02*E* − 01	1.56*E* − 01	7.62*E* − 01	7.78*E* − 01	4.07*E* − 01	2.73*E* − 01	1.71*E* − 01	6.00*E* − 01	7.84*E* − 01
hANN_PID	3.48*E* − 01	2.05*E* − 01	1.42**E** − 01	7.48*E* − 01	7.75*E* − 01	4.18*E* − 01	2.92*E* − 01	1.51**E** − 01	5.82*E* − 01	7.68*E* − 01
9-6-1										
sANN_MSE	3.80*E* − 01	2.32*E* − 01	2.11*E* − 01	7.54*E* − 01	7.45*E* − 01	4.41*E* − 01	3.16*E* − 01	2.21*E* − 01	6.31*E* − 01	7.50*E* − 01
sANN_tPI	3.74*E* − 01	2.30*E* − 01	1.93*E* − 01	7.56*E* − 01	7.48*E* − 01	4.51*E* − 01	3.31*E* − 01	2.10*E* − 01	6.13*E* − 01	7.37*E* − 01
sANN_PID	3.84*E* − 01	2.45*E* − 01	1.60*E* − 01	7.40*E* − 01	7.31*E* − 01	4.69*E* − 01	3.55*E* − 01	1.77*E* − 01	5.85*E* − 01	7.18*E* − 01
hANN_MSE	3.44*E* − 01	2.01*E* − 01	1.60*E* − 01	7.70*E* − 01	7.79*E* − 01	4.09*E* − 01	2.76*E* − 01	1.74*E* − 01	6.12*E* − 01	7.81*E* − 01
hANN_tPI	3.42**E** − 01	2.01*E* − 01	1.51*E* − 01	7.72**E** − 01	7.80*E* − 01	4.04*E* − 01	2.61**E** − 01	1.62*E* − 01	6.13*E* − 01	7.93**E** − 01
hANN_PID	3.45*E* − 01	2.02*E* − 01	1.45*E* − 01	7.64*E* − 01	7.78*E* − 01	4.15*E* − 01	2.87*E* − 01	1.58*E* − 01	6.14*E* − 01	7.72*E* − 01
9-8-1										
sANN_MSE	3.72*E* − 01	2.35*E* − 01	2.01*E* − 01	7.52*E* − 01	7.43*E* − 01	4.45*E* − 01	3.16*E* − 01	2.01*E* − 01	6.31*E* − 01	7.50*E* − 01
sANN_tPI	3.75*E* − 01	2.35*E* − 01	1.92*E* − 01	7.51*E* − 01	7.42*E* − 01	4.54*E* − 01	3.31*E* − 01	2.07*E* − 01	6.13*E* − 01	7.38*E* − 01
sANN_PID	3.76*E* − 01	2.38*E* − 01	1.75*E* − 01	7.48*E* − 01	7.39*E* − 01	4.65*E* − 01	3.49*E* − 01	1.87*E* − 01	5.92*E* − 01	7.23*E* − 01
hANN_MSE	3.43*E* − 01	1.98*E* − 01	1.56*E* − 01	7.70*E* − 01	7.83*E* − 01	3.95*E* − 01	2.62*E* − 01	1.68*E* − 01	6.32**E** − 01	7.92*E* − 01
hANN_tPI	3.44*E* − 01	1.94**E** − 01	1.62*E* − 01	7.67*E* − 01	7.87**E** − 01	3.94**E** − 01	2.61**E** − 01	1.68*E* − 01	6.05*E* − 01	7.93**E** − 01
hANN_PID	3.45*E* − 01	2.00*E* − 01	1.46*E* − 01	7.58*E* − 01	7.81*E* − 01	4.14*E* − 01	2.85*E* − 01	1.55*E* − 01	6.02*E* − 01	7.74*E* − 01
*Santa Ysabel Creek*										
9-4-1										
sANN_MSE	4.04*E* − 01	2.90*E* − 01	2.27*E* − 01	5.52*E* − 01	7.61*E* − 01	5.73*E* − 01	4.90*E* − 01	2.05*E* − 01	5.91*E* − 01	6.14*E* − 01
sANN_tPI	4.07*E* − 01	2.94*E* − 01	2.30*E* − 01	5.47*E* − 01	7.59*E* − 01	5.51*E* − 01	4.53*E* − 01	2.14*E* − 01	6.21**E** − 01	6.43*E* − 01
sANN_PID	4.17*E* − 01	3.11*E* − 01	1.91*E* − 01	5.20*E* − 01	7.44*E* − 01	6.38*E* − 01	6.01*E* − 01	1.80*E* − 01	4.98*E* − 01	5.26*E* − 01
hANN_MSE	3.65*E* − 01	2.53*E* − 01	1.89*E* − 01	5.77*E* − 01	7.92*E* − 01	4.99*E* − 01	3.83*E* − 01	1.77*E* − 01	5.40*E* − 01	6.98*E* − 01
hANN_tPI	3.59*E* − 01	2.56*E* − 01	1.92*E* − 01	5.80**E** − 01	7.90*E* − 01	4.63*E* − 01	3.52*E* − 01	1.81*E* − 01	5.43*E* − 01	7.22*E* − 01
hANN_PID	3.73*E* − 01	2.64*E* − 01	1.72**E** − 01	5.47*E* − 01	7.83*E* − 01	5.27*E* − 01	4.22*E* − 01	1.61**E** − 01	4.88*E* − 01	6.67*E* − 01
9-6-1										
sANN_MSE	4.05*E* − 01	2.91*E* − 01	2.40*E* − 01	5.51*E* − 01	7.61*E* − 01	5.69*E* − 01	4.91*E* − 01	2.27*E* − 01	5.89*E* − 01	6.12*E* − 01
sANN_tPI	3.99*E* − 01	2.88*E* − 01	2.46*E* − 01	5.57*E* − 01	7.64*E* − 01	5.52*E* − 01	4.64*E* − 01	2.23*E* − 01	6.12*E* − 01	6.34*E* − 01
sANN_PID	4.15*E* − 01	3.00*E* − 01	1.90*E* − 01	5.37*E* − 01	7.53*E* − 01	5.91*E* − 01	5.26*E* − 01	1.79*E* − 01	5.61*E* − 01	5.85*E* − 01
hANN_MSE	3.55*E* − 01	2.49*E* − 01	1.93*E* − 01	5.70*E* − 01	7.95*E* − 01	4.28**E** − 01	3.28**E** − 01	1.85*E* − 01	5.95*E* − 01	7.41**E** − 01
hANN_tPI	3.53*E* − 01	2.50*E* − 01	1.93*E* − 01	5.68*E* − 01	7.94*E* − 01	4.42*E* − 01	3.34*E* − 01	1.83*E* − 01	5.64*E* − 01	7.36*E* − 01
hANN_PID	−3.72*E* − 01	2.59*E* − 01	1.75*E* − 01	5.51*E* − 01	7.87*E* − 01	5.15*E* − 01	4.09*E* − 01	1.67*E* − 01	4.45*E* − 01	6.77*E* − 01
9-8-1										
sANN_MSE	4.08*E* − 01	2.92*E* − 01	2.66*E* − 01	5.49*E* − 01	7.60*E* − 01	5.65*E* − 01	4.82*E* − 01	2.39*E* − 01	5.97*E* − 01	6.20*E* − 01
sANN_tPI	4.03*E* − 01	2.88*E* − 01	2.54*E* − 01	5.57*E* − 01	7.64*E* − 01	5.51*E* − 01	4.60*E* − 01	2.29*E* − 01	6.16*E* − 01	6.37*E* − 01
sANN_PID	4.21*E* − 01	3.11*E* − 01	1.95*E* − 01	5.21*E* − 01	7.44*E* − 01	6.30*E* − 01	5.91*E* − 01	1.83*E* − 01	5.06*E* − 01	5.34*E* − 01
hANN_MSE	3.55*E* − 01	2.48**E** − 01	2.04*E* − 01	5.76*E* − 01	7.96**E** − 01	4.51*E* − 01	3.41*E* − 01	1.89*E* − 01	4.98*E* − 01	7.31*E* − 01
hANN_tPI	3.51**E** − 01	2.48**E** − 01	2.06*E* − 01	5.79*E* − 01	7.96**E** − 01	4.37*E* − 01	3.30*E* − 01	1.96*E* − 01	5.49*E* − 01	7.40*E* − 01
hANN_PID	3.65*E* − 01	2.57*E* − 01	1.78*E* − 01	5.32*E* − 01	7.89*E* − 01	5.06*E* − 01	3.96*E* − 01	1.69*E* − 01	3.30*E* − 01	6.88*E* − 01

**Table 10:** The values of PI2 on architectures 9-*N*_hd_-1 on SPEI forecast with hANN.

	Calibration period			Validation period		
	9-4-1	9-6-1	9-8-1	9-4-1	9-6-1	9-8-1
*Leaf River *						
9-4-1	0.00*E* + 00	−2.26*E* − 02	−2.00*E* − 02	0.00*E* + 00	−1.62*E* − 02	−3.61*E* − 02
9-6-1	2.21*E* − 02	0.00*E* + 00	2.51*E* − 03	1.59*E* − 02	0.00*E* + 00	−1.97*E* − 02
9-8-1	1.96*E* − 02	−2.51*E* − 03	0.00*E* + 00	3.49*E* − 02	1.93*E* − 02	0.00*E* + 00
*Santa Ysabel *						
9-4-1	0.00*E* + 00	−1.36*E* − 02	−1.56*E* − 02	0.00*E* + 00	−6.57*E* − 02	−9.33*E* − 03
9-6-1	1.34*E* − 02	0.00*E* + 00	−1.99*E* − 03	6.17*E* − 02	0.00*E* + 00	5.29*E* − 02
9-8-1	1.54*E* − 02	1.98*E* − 03	0.00*E* + 00	9.24*E* − 03	−5.59*E* − 02	0.00*E* + 00

**Table 11:** The values of PI2 on training functions for SPEI forecast with hANN.

	Calibration period			Validation period		
	MSE	tPI	PID	MSE	tPI	PID
*Leaf River *						
MSE	0.00*E* + 00	4.17*E* − 04	2.31*E* − 02	0.00*E* + 00	7.66*E* − 04	4.48*E* − 02
tPI	−4.17*E* − 04	0.00*E* + 00	2.27*E* − 02	−7.66*E* − 04	0.00*E* + 00	4.40*E* − 02
PID	−2.36*E* − 02	−2.32*E* − 02	0.00*E* + 00	−4.69*E* − 02	−4.61*E* − 02	0.00*E* + 00
*Santa Ysabel *						
MSE	0.00*E* + 00	−1.89*E* − 03	5.14*E* − 02	0.00*E* + 00	−1.73*E* − 02	1.81*E* − 01
tPI	1.89*E* − 03	0.00*E* + 00	5.32*E* − 02	1.70*E* − 02	0.00*E* + 00	1.95*E* − 01
PID	−5.42*E* − 02	−5.62*E* − 02	0.00*E* + 00	−2.22*E* − 01	−2.43*E* − 01	0.00*E* + 00
